# *C. spinosa* L. subsp. *rupestris* Phytochemical Profile and Effect on Oxidative Stress in Normal and Cancer Cells

**DOI:** 10.3390/molecules27196488

**Published:** 2022-10-01

**Authors:** Tiziana Bacchetti, Roberto Campagna, Davide Sartini, Monia Cecati, Camilla Morresi, Luisa Bellachioma, Erika Martinelli, Gabriele Rocchetti, Luigi Lucini, Gianna Ferretti, Monica Emanuelli

**Affiliations:** 1 Department of Life and Environmental Sciences, Polytechnic University of Marche, 60131 Ancona, Italy; 2 Department of Clinical Sciences, Polytechnic University of Marche, 60131 Ancona, Italy; 3 Department for Sustainable Food Process, Università Cattolica del Sacro Cuore, 29122 Piacenza, Italy; 4 New York-Marche Structural Biology Center (NY-MaSBiC), Polytechnic University of Marche, 60131 Ancona, Italy

**Keywords:** cancer, *Capperis spinosa* L. subsp. *rupestris*, glucosinolates, paraoxonase, polyphenols, oxidative stress

## Abstract

Spices, widely used to improve the sensory characteristics of food, contain several bioactive compounds as well, including polyphenols, carotenoids, and glucosynolates. Acting through multiple pathways, these bioactive molecules affect a wide variety of cellular processes involved in molecular mechanisms important in the onset and progress of human diseases. *Capparis spinosa* L. is an aromatic plant characteristic of the Mediterranean diet. Previous studies have reported that different parts (aerial parts, roots, and seeds) of *C. spinosa* exert various pharmacological activities. Flower buds of *C. spinosa* contain several bioactive compounds, including polyphenols and glucosinolates. Two different subspecies of *C. spinosa* L., namely, *C. spinosa* L. subsp. *spinosa*, and *C. spinosa* L. subsp. *rupestris*, have been reported. Few studies have been carried out in *C. spinosa* L. subsp. *rupestris*. The aim of our study was to investigate the phytochemical profile of floral buds of the less investigated species *C. spinosa* subsp. *rupestris*. Moreover, we investigated the effect of the extract from buds of *C. spinosa* subsp. *rupestris* (CSE) on cell proliferation, intracellular ROS levels, and expression of the antioxidant and anti-apoptotic enzyme paraoxonase-2 (PON2) in normal and cancer cells. T24 cells and Caco-2 cells were selected as models of advanced-stage human bladder cancer and human colorectal adenocarcinoma, respectively. The immortalized human urothelial cell line (UROtsa) and human dermal fibroblast (HuDe) were chosen as normal cell models. Through an untargeted metabolomic approach based on ultra-high-performance liquid chromatography quadrupole-time-of-flight mass spectrometry (UHPLC-QTOF-MS), our results demonstrate that *C. spinosa* subsp. *rupestris* flower buds contain polyphenols and glucosinolates able to exert a higher cytotoxic effect and higher intracellular reactive oxygen species (ROS) production in cancer cells compared to normal cells. Moreover, upregulation of the expression of the enzyme PON2 was observed in cancer cells. In conclusion, our data demonstrate that normal and cancer cells are differentially sensitive to CSE, which has different effects on PON2 gene expression as well. The overexpression of PON2 in T24 cells treated with CSE could represent a mechanism by which tumor cells protect themselves from the apoptotic process induced by glucosinolates and polyphenols.

## 1. Introduction

Several epidemiological studies have indicated that dietary intake of vegetable bioactive molecules exerts a key role in prevention of several chronic diseases [1]. Among vegetables, spices, commonly used to improve the appeal and sensory characteristics of food, contain several bioactive compounds, including polyphenols, carotenoids, and glucosynolates [2]. These bioactive molecules act through multiple pathways to affect a wide variety of cellular processes involved in cancer, including differentiation, proliferation, apoptosis, and cell-cycle arrest. In addition to their ability to act as antioxidants, they regulate cell functions and modulate gene expression [2,3,4,5,6]. Pro-oxidant and cytotoxic effects of polyphenols have been described under certain conditions and in certain tissues, including in tumour cells [7,8,9,10,11].

*Capparis spinosa* L. is an aromatic plant widespread in Mediterranean Europe. The flower buds (capers) are used as a food ingredient in Mediterranean cuisine. Previous studies have reported that capers are a good source of flavonoids (rutin, kaempferol), phenolic acids, and glucosinolates (glucocapparin, glucoiberin, sinigrin, glucobrassicin) which are known to provide health-improving benefits due to their biological activities (antioxidant, anticancerogenic, antimicrobial, antimutagenic) [12,13,14,15,16]. Previous studies have assessed the effect of the *C. spinosa* extract on different cancer cell lines. Hydroalcoholic extract of *C. spinosa* (floral buds and leaves) decreased growth and proliferation in different cancer cell lines such as cervical cancer (Hela), breast cancer (MCF7), and osteosarcoma (Saos-2) compared to normal cells (Fibroblast) [17]. *C. spinosa* essential oil and aqueous infusion (floral buds and leaves) showed an inhibitory effect on colorectal adenocarcinoma cell (HT-29) proliferation [18].

Two different subspecies of *C. spinosa* L, namely, *C. spinosa* L. subsp. *spinosa* and *C. spinosa* L. subsp. *rupestris* (syn. *C. orientalis*), have been reported [19,20,21]. Few studies have been carried out on *C. spinosa* L. subsp. *rupestris*. Studies investigating seeds, leaves [19], and flower buds [20,22] of *C. spinosa* L. subsp. *rupestris* have reported that it represents a very rich source of bioactive compounds of nutraceutical relevance, including fatty acids, glucosinolates, and polyphenols, as does the more widely studied subspecies, *C. spinosa* subsp. *spinosa*.

The aim of our study was to further investigate the phytochemical profile of the floral buds of the less investigated subspecies, *C. spinosa* subsp. *rupestris,* through an untargeted metabolomic approach based on ultra-high-performance liquid chromatography quadrupole-time-of-flight mass spectrometry (UHPLC-QTOF-MS), focusing on polyphenols and glucosinolates. Moreover, we explored antioxidant activity and the effect of the extract of buds of *C. spinosa* subsp. *rupestris* in both normal and cancerous cells. Human bladder carcinoma cells (T24) and human colorectal adenocarcinoma cells (Caco-2) were selected as cancer cell models. The immortalized human urothelial cell line (UROtsa), which is considered to be a valuable model for normal human bladder urothelium [23,24,25], along with human dermal fibroblast (HuDe), were included as normal cell models. We investigated the modulatory role of the extract on cell proliferation, intracellular ROS levels, and expression of the antioxidant and anti-apoptotic intracellular enzyme paraoxonase-2 (PON2) [26,27,28,29,30], which has been reported to be upregulated in different human cancers and to play a role in the physiopathology of cancer [26,31,32,33,34]. 

## 2. Results

### 2.1. Phytochemical Profile of C. spinosa *subsp.* rupestris Floral Buds 

The untargeted metabolomics approach based on UHPLC-ESI/QTOF-MS allowed us to putatively annotate 428 compounds in *C. spinosa* subsp. *rupestris* extract (CSE). A comprehensive list reporting all the identified compounds, together with their abundance and composite mass spectrum, can be found in the Supplementary Materials. In detail, 363 were polyphenols and 65 were glucosinolates. Among polyphenols, the class with the most abundant compounds was that of flavonoids (173 compounds), characterized by 52 flavonols, 48 anthocyanins, 11 flavanols, and 62 other flavonoids (e.g., flavones). Furthermore, 82 phenolic acids were identified, along with 26 lignans, 10 stilbenes, and 72 other low-molecular-weight phenolics (quantified as tyrosol equivalents).

In this regard, Table 1 displays the results from the semi-quantitative analysis. A cumulative phenolic content of 1.65 g equivalents (Eq.)/100 g dry matter (DM) was revealed. The most abundant phenolic class belonged to low-molecular-weight phenolics (tyrosol derivatives; 0.86 ± 0.16 g Eq./100 g DM), followed by flavonoids (0.49 g Eq./100 g DM). In particular, flavonoids consisted of anthocyanins (0.28 ± 0.02 g Eq./100 g DM), flavonols (0.08 ± 0.01 g Eq./100 g DM), flavanols (0.04 ± 0.00 g Eq./100 g DM), and other flavonoids (0.09 ± 0.00 g Eq./100 g DM). Therefore, anthocyanins were highlighted as the most abundant sub-class among flavonoids. The phenolic profile of CSE extract was characterized by 0.19 ± 0.04 g Eq./100 g DM of lignans, 0.09 ± 0.01 g Eq./100 g DM of phenolic acids, and 0.03 ± 0.00 g Eq./100 g DM of stilbenes. Finally, glucosinolates were quantified in CSE as gluconapin equivalents; a total content of 14.23 ± 1.01 g/100 g DM was detected. In particular, the highest abundance values was recorded for glucocapparin. 

Results are expressed as mean values (g phenolic equivalents) ± standard deviation (*n* = 3) in 100 g dry matter (DM). Eq. = Equivalents (cyanidin for anthocyanins, quercetin for flavonols, luteolin for flavones and other flavonoids, catechin for flavan-3-ols, ferulic acid for phenolic acids, sesamin for lignans, resveratrol for stilbenes, tyrosol for tyrosols and other remaining phenolics and gluconapin for glucosinolates).

### 2.2. Antioxidant Properties of C. spinosa *subsp.* rupestris Floral Buds

Total polyphenolic (TP) content in CSE was 1.6 ± 0.1 g gallic acid equivalent (GAE)/100 g DM, in agreement with the cumulative phenolic content obtained by the semi-quantitative analysis. The in vitro antioxidant capacity of CSE as evaluated by Oxygen Radical Scavenging Capacity (ORAC) assay was found to be 50.8 ± 8.4 mmol Trolox Equivalent (TE)/100 g DM. Pearson’s correlation analysis allowed us to detect significant correlations between levels of flavan-3-ols (r = 0.999; *p* < 0.01) and phenolic acids (r = 0.998; *p* < 0.01) and ORAC antioxidant activity. Neither the levels of other phenolic classes nor the total content of glucosinolates were significantly correlated with ORAC values.

### 2.3. Effect of C. spinosa rupestris on Cell Proliferation

**Figure 1:**
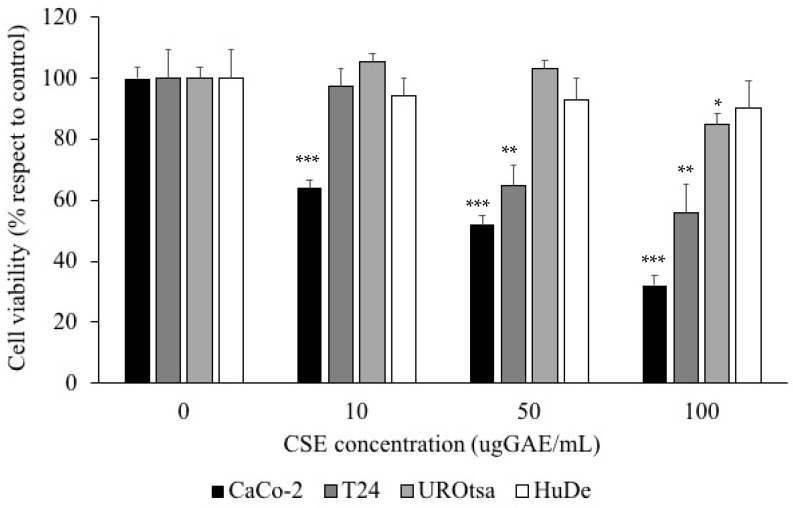
The effect of *C. spinosa* subsp. *rupestris* extract (CSE) polyphenols on cell proliferation in human colorectal adenocarcinoma cells (Caco-2) (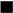), human bladder carcinoma cells (T24) (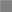), immortalized human urothelial cell line (UROtsa) (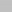), and human dermal fibroblast (HuDE) (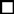). All values are expressed as mean ± standard deviation. All reported values are expressed as mean ± standard deviation. *** Caco-2 vs. Caco-2 + CSE; ** T24 vs. T24+CSE; * UROtsa vs. UROtsa + CSE (ANOVA: *: *p <* 0.05; **: *p* < 0.005; ***: *p* < 0.001).

The effect of CSE on cell proliferation was evaluated on cancer cells (Caco-2 and T24) and normal cells (UROtsa and HuDe) at different TP concentrations (0,10, 50 and 100 µg GAE/mL). As shown in Figure 1, CSE exhibited a higher anti-proliferative effect in Caco-2 cells and in T24 cells (Figure 1) compared with UROtsa and HuDe cells (Figure 1). In particular, a significant decrease of cell viability was observed in Caco-2 cells treated with the lowest CSE concentration (10 µg GAE/mL) (64.2 ± 2.7% with respect to control). Significant differences were observed at the highest concentrations as well (*p* < 0.001).

A significant decrease in cell viability was observed in T24 cells starting from 50 ug GAE/mL (65.1 ± 6.8% respect to control) (*p* < 0.005) (Figure 1). A significant effect of CSE on proliferation of UROtsa cells was evident only at the highest concentration of CSE (100 ug GAE/mL) (85.2 ± 2.9% respect to control) (*p* < 0.05). No effect of CSE was observed in HuDe cells (Figure 1). 

### 2.4. Effect of C. spinosa Extract on Intracellular Reactive Oxygen Species Levels

The levels of intracellular reactive oxygen species (ROS) evaluated using a 2′,7′-dichlorodihydrofluorescein diacetate (H_2_DCFDA) fluorescent probe showed that the Caco-2 cell line had the highest significant baseline levels (0.83 ± 0.01 a.u). Lower levels of ROS were demonstrated in T24 cells (0.73 ± 0.02 a.u). Both UROtsa and and HuDe cells had comparable levels of ROS (0.48 ± 0.01 a.u and 0.51 ± 0.02 a.u). 

**Figure 2:**
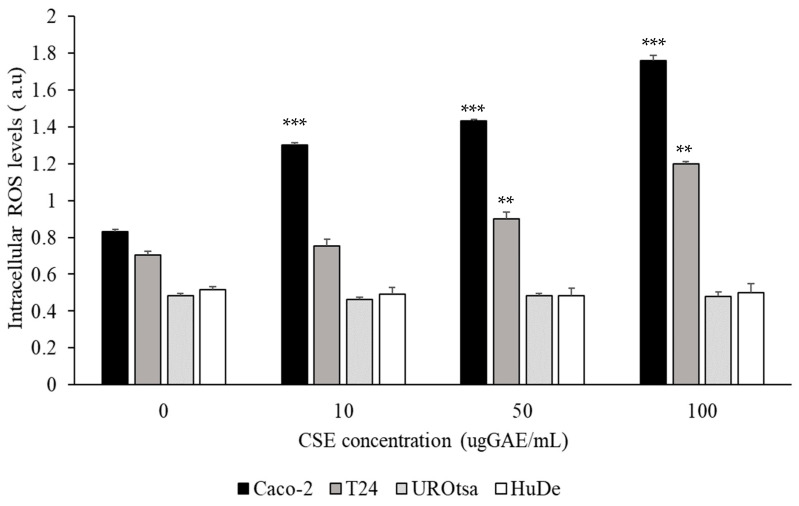
Effect of CSE polyphenols on intracellular reactive oxygen species (ROS) production in cancer cell lines Caco-2 (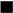) and T24 (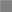) and normal cell lines UROtsa (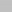) and HuDE (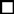). ROS levels were determined after 48 h of incubation. All values are expressed as mean ± standard deviation. All reported values are expressed as mean ± standard deviation *** Caco-2 vs. Caco-2 + CSE; ** T24 vs. T24 + CSE(ANOVA: **: *p* < 0.005; ***: *p* < 0.001).

The effect of CSE on intracellular ROS levels in normal and cancer cells was studied at different TP concentrations (0,10, 50 and 100 µg GAE/mL). No modification of ROS levels was observed in cell lines during incubation for 48 h in the absence of extracts (data not shown). As shown in Figure 2, treatment with CSE resulted in a significant increase of intracellular ROS levels in Caco-2 and T24 cells in a dose-dependent manner. In Caco-2 cells treated with the lowest CSE concentration (10 µg GAE/mL) an increase of intracellular level of ROS (about 56%) was observed with respect to basal levels (*p* < 0.001). In T24 cells, the increase was statistically significant in cells treated with CSE 50 µg GAE/mL (about 24% compared to baseline) (*p* < 0.005). The levels of intracellular ROS were not significantly modified in UROtsa and HuDe treated with increasing concentrations of CSE compared to untreated cells (Figure 2). 

### 2.5. Effect of C. spinosa *subsp.* rupestris on PON2 Expression 

**Figure 3:**
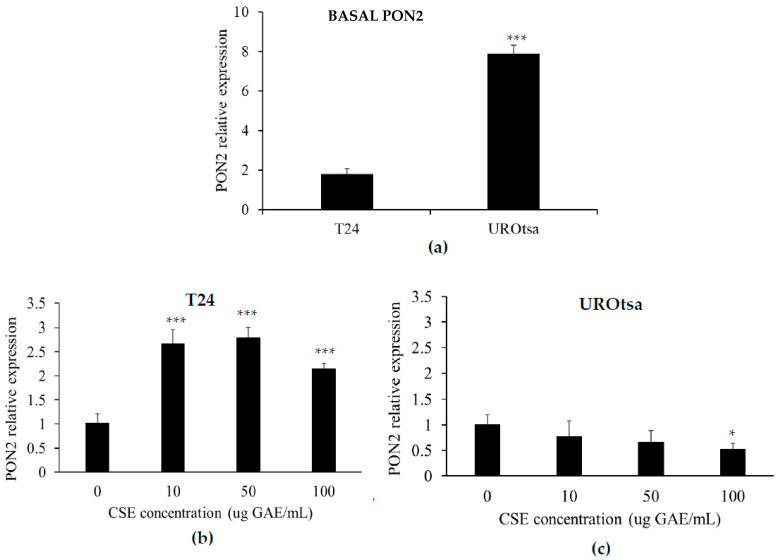
Effect of CSE polyphenols on paraoxonase-2 (PON2) expression. Real-Time PCR was used to evaluate (**a**) mRNA levels in T24 and UROtsa cells (***: *p* < 0.001); (**b**) modifications of PON2 mRNA levels in T24 cells treated with CSE for 48 hours (T24 vs. T24 + CSE, ***: *p* < 0.001; and (**c**) modifications of PON2 mRNA levels in UROtsa cells treated with CSE for 48 hours (UROtsa vs. UROtsa + CSE, *: *p* < 0.05). All reported values are expressed as mean ± standard deviation.

To better investigate the effect of CSE on normal and cancer cells, the expression levels of the antioxidant and antiapoptotic enzyme PON2 were analyzed in T24 and UROtsa cells. The relative baseline expression levels of PON2 were significantly lower in the T24 cells compared to the UROtsa (*p* < 0001); PON2 expression in UROtsa cells was about four times higher compared to T24 cells (Figure 3a).

Incubation of CSE on UROtsa cells was associated with opposite effects with respect to T24. A significant increase in PON2 expression was observed in T24 cells after incubation with CSE at all tested concentrations (Figure 3b) (*p* < 0.001). PON2 expression was about 2.5-time higher in T24 cells treated with the lower CSE concentration (10 µg GAE/mL) compared to untreated cells; no further increase was observed in cells treated with higher CSE concentrations (Figure 3b). On the contrary, treatment with CSE triggered a slight downregulation of PON2 in UROtsa cells; the effect was significant at higher concentrations as well (100 µg GAE /mL) (*p* < 0.05) (Figure 3c).

## 3. Discussion

*C. spinosa L.* has been used in folk medicine since ancient times for its numerous biological activities [16,35]. Two different subspecies of *C. spinosa* L., namely, *C. spinosa* L. subsp. *spinosa* and *C. spinosa* L. subsp. *rupestris* (syn. *C*. *orientalis*), have been reported [19,20,21]. Few studies have been carried out using *C. spinosa* L. subsp. *rupestris* [19,20,22]. 

In this study, an untargeted metabolomic approach was applied to investigate the phytochemical profile of *C. spinosa* subsp. *rupestris* flower bud extract. The phenolic composition of *C. spinosa* flower buds has been extensively investigated in the past years by different analytical methods [15,36,37,38]. Our results from untargeted profiling demonstrate that *C. spinosa* subsp. *rupestris* flower buds are a good source of flavonoids; in particular, different glycosylated forms of quercetin, kaempferol, myricetin, and isorhamnetin derivatives were identified. Moreover, rutin (quercetin-3-*O*-rutinoside) has been reported as the most abundant flavonoid [15,36,37,38].

In addition, hydroxycinnamic acids, hydroxybenzoic acids, and flavan-3-ols have been detected in this food matrix [15,39]. Among anthocyanins, pelargonidin 3-*O*-glucoside and pelargonidin 3-*O*-rutinoside have been identified [37]. Our semi-quantitative data reveal that C. *spinosa* L. subsp. *rupestris* was particularly rich in low-molecular-weight phenolics and flavonoids, accounting for a total phenolic content of 1.65 g equivalents/100 g DM. The levels of total phenols are similar to those recently reported by Grimalt et al. (2021) in flower buds of C. *spinosa* L. subsp. *rupestris* [22]. However, quantitative data are hardly comparable with the available literature, as the content of these bioactive compounds is strictly dependent on genetic factors, growth stage, agronomical conditions, extraction, and analytical methods as well [15,35]. 

Thanks to its close relation to the Brassicaceae family, *C. spinosa* is an important source of glucosinolates, secondary plant metabolites known for their role in preventing disease and reducing the risk of carcinogenesis [16]. The literature data were confirmed by our untargeted metabolomics approach, revealing a high content of glucosinolates (14.23 ± 1.01 g/100 g DM). Glucocapparin was the most abundant glucosinolate detected in our experimental conditions, in agreement with previous data [37,40,41]. A significant correlation was established between both flavan-3-ols (r = 0.999; *p* < 0.01) and phenolic acids (r = 0.998; *p* < 0.01) and antioxidant activity, as evaluated through ORAC assay. No significant correlation was detected when considering the other phenolic classes or the total content of glucosinolates, suggesting that flavanols are the major determinant of the in vitro antioxidant activity of CSE.

Several studies have shown that bioactive phytocompounds affect cancer cell growth, differentiation, and apoptosis [9,10,17]. At the molecular level, many studies have shown that their action can be attributed to their ability to modulate oxidative damage as well as to their capacity to interact with basic cellular and endogenous antioxidant mechanisms [10,42,43]. 

Therefore, in the present study we investigated the effect of CSE on cell proliferation, intracellular ROS levels, and PON2 expression in normal and cancer cells. T24 cells, a model of advanced-stage human bladder cancer with a high capacity for proliferation and metastasis, and human colorectal adenocarcinoma (Caco-2) cells were selected as cancer cell models. The immortalized human urothelial cell line UROtsa, which is considered to be a valuable model for normal human bladder urothelium [23,24,25], and human dermal fibroblast (HuDe) were chosen as normal cell models.

Our analysis of the effects of polyphenol-rich extract from *C. spinosa* subsp. *rupestris* (CSE) on cell proliferation and intracellular ROS levels shows significant differences between cancer cells (Caco-2 and T24) and normal cells (UROtsa and HuDE). We observed a higher anti-proliferative effect exerted by CSE polyphenols in Caco-2 and T24 cells compared to the normal UROtsa and HuDe cell lines. 

In order to assess the cytotoxicity mechanism produced by CSE on cancer cells, this study investigated the effect on oxidative stress. A CSE polyphenol concentration-dependent increase of ROS was observed in cancer cells. On the contrary, no significant effect of CSE polyphenols on intracellular ROS was observed in normal cells. In addition, our study reports differences in the expression of the antioxidant and antiapoptotic enzyme PON2 after incubation with CSE. A significant increase was observed in T24; on the contrary, treatment with CSE triggered a significant downregulation of PON2 in UROtsa cells.

Our results are in agreement with Moghadamnia et al., who reported that hydroalcoholic extract of *C. spinosa* (floral buds and leaves) was able to decrease growth and proliferation in different cancer cell lines (Hela, MCF7, and Saos-2) and had a low effect on fibrostast cells used as control cells [17]. These data are confirmed by other studies showing non-malignant cells to be less sensitive to the inhibitory growth effect of polyphenols such as apigenin [44] and curcumol [45] compared with cancer cell lines.

As concerns the mechanisms potentially involved in CSE-induced ROS production and PON2 regulation in cancer cells, the literature data suggest that, although polyphenols are widely studied for their antioxidant properties in vitro, certain molecules can exert a pro-oxidant effect [7,8,9,10,11]. Redox activity in biological systems, metal ions, pH, and oxygen levels are all involved, and can affect the auto-oxidation kinetics of polyphenols. More recently, Kanner et al. [46] reviewed the role of polyphenols and the generation of H_2_O_2_ and confirmed their physiological relevance both in vitro and in vivo. In agreement with the literature data, we suggest that the polyphenol-mediated peroxide generation could be involved in the cytotoxic effect on Caco-2 and T24 cancer cells. 

It has been widely demonstrated that glucosinolate hydrolysis products, namely, isothiocyanates, exert anti-carcinogenic activity by inducing apoptosis of cancer cells. While the mechanisms of action are not yet fully understood, different potential underlying mechanisms through which glucosinolate derivatives modify the apoptotic pathway, thereby leading to controlled proliferation and cancer cell death, have been suggested [47,48]. It has previously been reported that dietary isothiocyanates can induce apoptosis and/or arrest cell-cycle progression in human bladder carcinoma lines (UM-UC-3 and T24) [49]. According to our findings as well as those of several other studies, exposure to glucosinolate derivatives can lead to reduced cell proliferation and increase ROS in a dose-dependent manner in several cancer cells, including cervical cancer cells [50], colon cancer cells (Caco-2 and HT29) [51,52], prostatic adenocarcinoma cells (PC-3) [53], osteosarcoma cells (LM8) [54], and lung adenocarcinoma cells (LTEP-A2) [55]. Interestingly, Trachootham et al. [56], have revealed that normal and cancer cells respond differently to treatment with β-phenylethyl isothiocyanate due to differences in their redox states. Particularly, this glucosinolate derivative led to an abnormal increase in ROS only in malignant cells, which was due to their intrinsically higher oxidative stress levels compared to normal cells. Therefore, the higher pro-oxidant and cytotoxic effect of polyphenols and glucosinolates in cancer cells might be related to metabolic factors and to the higher concentrations of H_2_O_2_ produced in tumor cells compared to corresponding normal cells [57]. The direction of many cellular processes and reactions depends on the intracellular “redox state”. In our experimental conditions, higher baseline levels of intracellular ROS were observed in cancer cells (Caco-2 and T24) compared to normal cells (UROtsa and HuDE). These data are in agreement with previous studies demonstrating that ROS are a key determinant of cancer’s metabolic phenotype and that cancer cells are characterized by enhanced metabolic activity, resulting in changes in the cellular redox state to handle the production of high levels of ROS [58,59,60]. Signaling pathways activated by an increase in intracellular H_2_O_2_ include the redox-sensitive antioxidant response element (ARE) [61] and the JNK/AP-1 pathway [62]. Both pathways can cause increased expression of antioxidant enzymes, including PON2 [63]. Therefore, overproduction of ROS suggests a higher dependence of cancer cells on the antioxidant system to maintain redox balance, which could explain differences in relative PON2 expression among T24 and UROtsa cells.

The differences in the effects of CSE on PON2 expression in T24 cancer cells and UROtsa could be related to different mechanisms, including intracellular localization of the enzyme and intracellular oxidative balance. In fact, differences in PON2 localization have been described in different cells and/or tissues. Indeed, in addition to the endoplasmic reticulum, PON2 appears to localize in the mitochondria, plasma membrane, and nucleus [30,64]. The cell type in which PON2 is expressed may influence the localization of PON2 protein and its biological activity. Among factors likely related to the differences in modulation of PON2 expression in T24 and UROtsa cells, a relationship between oxidative balance and signaling pathways can be proposed [31,65]. According to Shiner et al., PON2 expression is dose-dependently up-regulated by pomegranate juice phenolics in mouse monocyte-macrophage (J774A.1) via peroxisome proliferator-activated receptor gamma (PPARγ) and activator protein-1 (AP-1) pathway activation [63]. The authors showed that punicalagin and gallic acid were potent upregulators of PON2 [63]. Both these phenolic compounds were detected in CSE. An increase in PON2 relative gene expression has been observed in THP-1 macrophages incubated with chlorogenic acid. In contrast, a decrease in PON2 expression was observed at higher polyphenol concentrations [66]. This latter trend suggests that polyphenol modulation of PON2 expression is strictly related to structural differences among different compounds and different concentrations. 

Whatever the mechanisms involved in the upregulation of PON2 expression in CSE-incubated T24 cells, several hypotheses can be advanced concerning the physiological consequences. The increase in PON2 expression could represent a mechanism to compensate for the enhancement in oxidative stress. An increase in PON2 gene expression and activity has been observed, for instance in response to oxidative stress in both ex vivo and in vivo models. In detail, PON2 was upregulated in response to oxidative stress in different cell types (HepG2 cells and macrophages) and animal models (mice fed high fat diets and ApoE knockout mice) [66,67,68,69]. An increase in PON2 protected cells against oxidative stress induced by oxidants such as H_2_O_2_ and 2,3-dimethoxy-1,4-naphthoquinone (DMNQ) and significantly diminished their toxicity [69]. Our own previous studies have demonstrated that intracellular ROS production triggered by tert-butyl hydroperoxide was significantly lower in PON2-overexpressing T24 cells than control cells [70]. Moreover, in the same cell model, PON2 expression modulated susceptibility to the chemotherapeutic agents cisplatin and gemcitabine [71]. 

As previously mentioned, CSE extract contains several different bioactive molecules, including polyphenols and glucosinolates. These molecules act through multiple pathways, and each ingredient can have different molecular targets. At present, we suggest that an additive as well as synergistic interaction between several phytochemicals could contribute to the effect of CSE on cell proliferation, ROS levels, and PON2 modulation.

## 4. Materials and Methods

### 4.1. Extraction of Polyphenols and Glucosinolates and Untargeted UHPLC-QTOF Profiling

The *C. spinosa* subsp. *rupestris* plants were grown in Borgo Cisterna (Santa Lucia Cisterna, Macerata Feltria, PU Italy). Flower buds of *C. spinosa* were washed, frozen at −20 °C, freeze-dried, and shredded.

The untargeted profiling of the powdered *Capparis spinosa* samples was depicted by ultra-high-performance liquid chromatography quadrupole-time-of-flight mass spectrometry (UHPLC-ESI/QTOF-MS). Powdered samples were processed through ultrasonic-assisted extraction using 0.1% formic acid in 80% methanol as the extraction solvent (1:10 *w*/*v*). After centrifugation (7830× *g*, 10 min, 4 °C), the supernatant was transferred to an HPLC vial for analysis. Previously optimized analytical conditions were followed [72]. In this regard, a C18-column Agilent Zorbax Eclipse Plus (50 mm × 2.1 mm, 1.8 μm) and a water–acetonitrile binary gradient (6% acetonitrile to 94% acetonitrile in 32 min) were used for chromatographic separation. Mass spectrometry was carried out in positive full-scan mode, acquiring ions in the range 100–1200 *m*/*z* (scan rate: 0.8 spectra/s, mass resolution 30,000 FWHM). The injection volume was 6 μL, and three replications were analyzed together with a random injection of blank samples (i.e., solvent only). Agilent Profinder B.07 software was then used to process the raw mass features, according to the ‘find-by-formula’ algorithm and considering the whole isotope pattern (i.e., monoisotopic mass, isotopic spacing, and isotopic ratio). Phenol-Explorer 3.6 and a custom database for glucosinolates were used for putative annotation [73]. Accordingly, an additional level of confidence in the annotation was achieved using a typical tolerance of 5 ppm for mass accuracy when considering QTOF instruments. Post-acquisition data filtering, baselining, and normalization were performed using Agilent Mass Profiler Professional B.12.06 software by retaining only those compounds identified within 100% of replications within at least one treatment. For the semi-quantitative analysis, the isobaric compounds were removed from the raw metabolomic dataset. Afterwards, the phenolic compounds and glucosinolates were classified into classes and then quantified using pure reference compounds (purity > 98%) representative of each respective class (i.e., cyanidin for anthocyanins, quercetin for flavonols, luteolin for flavones and other flavonoids, catechin for flavan-3-ols, ferulic acid for phenolic acids, sesamin for lignans, resveratrol for stilbenes, tyrosol for tyrosols and other remaining phenolics, and gluconapin for glucosinolates). The results were expressed as g equivalents (Eq.)/100 g DM. 

### 4.2. Assessment of Total Phenolic Content and Antioxidant Activity

Total polyphenolic (TP) content was evaluated in extracts obtained from *C. spinosa* subsp. *rupestris* by Folin–Ciocalteu assay [74]. TP levels in CSE were expressed as grams of gallic acid equivalent (GAE) per 100 g of dry matter (g GAE/100 g DM). 

The total antioxidant capacity (TAC) of CSE was determined by oxygen radical absorbance capacity (ORAC) assay using fluorescein as the fluorescent probe and 2,2′-azobis (2-methylpropionamide) dihydrochloride (AAPH) as the oxidizing agent [75]. Trolox (from 5 to 300 µM) was used to calibrate the assay. Fluorescence emission intensity was recorded every 5 min for 3 h at λ ex 485/λ em 530 nm in a Multi-Mode Microplate Reader SynergyTM HT (BioTek Instruments, Inc., Winooski, VI, USA). A blank sample containing PBS, Fluorescein, and AAPH was prepared. The final ORAC values were calculated using the net area under the curve (AUC) of decay. Results were expressed as 

Trolox equivalents per 100 g of dry matter (mmol TE/100 g DM) and data were presented as mean values ± standard deviation of triplicate independent experiments.

### 4.3. Cell Culture and Treatment 

Human bladder carcinoma T24 cells (ATCC^®^ HTB-4™), human colorectal adenocarcinoma (Caco-2 cells) (ATCC^®^HTB-37™), and human dermal fibroblast (HuDe) (ATCC^®^PCS-201-012™) cells were obtained from the American Type Culture Collection (ATCC, Rockville, MD, USA), while SV-40 transformed human bladder urothelial cell line (i.e., UROtsa cell line) was a kind gift from Dr. Scott H. Garrett (Department of Urology, West Virginia University, Morgantown, WV, USA)

T24 cells and UROtsa Cells were cultured in DMEM/F12 medium, while Caco-2 cells and HuDe cells were cultured in DMEM and MEM medium, respectively. Both media were supplemented with 10% fetal bovine serum (FBS) and gentamicin 50 µg/mL. All cells were maintained under standard tissue culture conditions of 37 °C, 95% air/5% CO_2_.

### 4.4. Cell Viability Assay

Cell viability was assessed through a colorimetric assay with 3-(4,5-dimethylthiazol-2-yl)-2,5-diphenyl tetrazolium bromide (MTT) [76]. Briefly, cells were seeded in 96-well plates (5 × 10^3^ cells/well) and allowed to attach overnight. The cells were treated with CSE containing increasing concentrations of polyphenols (0,10, 50 and 100 µg GAE/mL) for 48 h. After 48 h of incubation with CSE, a volume of 10 μL of MTT reagent (5 mg/mL in phosphate buffered saline) was added to each well and incubated for 4 h at 37 °C. The medium was then removed and 200 μL isopropanol was added. As an indicator of living and metabolically active cells, the degree of formazan formation was measured at 570 nm. Results were expressed as a percentage with respect to control (untreated cells) and presented as mean values ± standard deviation of three independent experiments performed in triplicate.

### 4.5. Detection of Intracellular ROS Levels

A 2′,7′-dichlorodihydrofluorescein diacetate (DCFH_2_-DA) probe (Sigma-Aldrich, St. Louis, MO, USA) was used to detect the levels of intracellular reactive oxygen species in cells treated under different experimental conditions. A stock solution of DCFH_2_-DA was prepared in DMSO. Cells were seeded on 96-well black plates (5 × 10^3^ cells per well) and allowed to adhere overnight. The cells were incubated with increasing concentrations (0, 10, 50, and 100 µg GAE/mL) of CSE for 48 h. At the end of the incubation period, the medium was removed and the cells were pre-treated for 45 min at 37 °C with DCFH_2_-DA (50 μM) in the dark. A volume of the probe was then added from the stock solution. Cells were washed to remove extracellular DCFH_2_-DA and then phosphate-buffered saline was added. The fluorescence of the cells from each well was measured and recorded on a fluorescence plate reader at λex/λem (485/535 nm) (Multi-Mode Microplate Reader SynergyTM HT, BioTek Instruments, Inc.) [71]. Results were presented as mean values ± standard deviation of independent experiments performed in triplicate.

### 4.6. Real-Time PCR 

Cell pellets (1 × 10^6^ cells) were homogenized in a lysis buffer and total RNA was isolated with SV Total RNA Isolation System (Promega, Madison, WI, USA) according to the manufacturer’s protocol. the quantity and quality of RNA were spectrophotometrically evaluated at 260 and 280 nm. Total RNA (2 μg) was reverse transcribed with M-MLV Reverse Transcriptase (Promega) using random primers in a total volume of 25 μL for 60 min at 37 °C.

To examine PON2 gene (NG_008725) expression quantitatively in T24 and UROtsa cells treated under different experimental conditions, Real-Time PCR analyses were performed using a CFX96 Real-Time PCR Detection System (Bio-Rad Laboratories, Hercules, MA, USA); the cDNA generated as described above was utilized as a template. To avoid false positive results caused by amplification of contaminating genomic DNA during cDNA preparation, all primers were selected to flank an intron. PCR efficiency was tested for both primer pairs and found to be close to 1. The primers used were (forward) 5′-TCGTGTATGACCCGAACAATCC-3′ and (reverse) 5′-AACTGTAGTCACTGTAGGCTTCTC-3′ for PON2, and (forward) 5′-TCCTTCCTGGGCATGGAGT-3′ and (reverse) 5′-AGCACTGTGTTGGCGTACAG-3′ for β-actin. Genes were run in duplicate utilizing SsoFastEvaGreenSupermix (Bio-Rad Laboratories, USA) according to the following protocol: 40 cycles at 95 °C for 30 s and 58 °C for 30 s. Samples were assayed in triplicate utilizing β-actin gene for data normalization. The expression level of PON2 in both treated and untreated cells was expressed as the fold change and calculated by 2^−ΔΔCt^, where ΔCt = Ct (PON2) − Ct (β-actin) and ΔΔCt = ΔCt (treated cells) − ΔCt (untreated cells) [71].

### 4.7. Statistical Analysis

Data were analyzed using IBM SPSS Statistics version 27 (IBM Corporation, New York, NY, USA). Significant differences between groups were determined using the Mann–Whitney U test. A *p*-value < 0.05 was considered statistically significant. The Pearson’s correlation coefficients (0.01< *p* < 0.05) between the untargeted phytochemical profiling and the different spectrophotometric assays (i.e., TP and ORAC) were evaluated using the same statistical software.

## 5. Conclusions

In conclusion, we are able to confirm that *C. spinosa rupestris* flower buds contain polyphenols and glucosinolates able to exert a higher cytotoxic effect in cancer cells compared to normal cells. Among the mechanisms which explain the role of polyphenols are generation of H_2_O_2_ and effects on cell redox signaling. The overexpression of PON2 in T24 cells treated with CSE phytochemicals might be related to the increase of intracellular ROS, and could represent a mechanism used by tumor cells to protect themselves from the apoptotic process induced by glucosinolates and polyphenols.

## Figures and Tables

**Table 1:** Semi-quantitative data of polyphenols (classes/subclasses) and glucosinolates identified from untargeted ultra-high-performance liquid chromatography quadrupole-time-of-flight mass spectrometry (UHPLC-QTOF-MS) profiling.

Class (g Eq./100 g)	*C. spinosa* subsp. *rupestris* Floral Buds
Anthocyanins	0.28 ± 0.02
Flavonols	0.08 ± 0.01
Flavanols	0.04 ± 0.00
Other flavonoids	0.09 ± 0.00
Phenolic acids	0.09 ± 0.01
Lignans	0.19 ± 0.04
Stilbenes	0.03 ± 0.00
Other phenolics	0.86 ± 0.16
Glucosinolates	14.23 ± 1.01

## Data Availability

Not applicable.

## Supplementary Materials

The following supporting information can be downloaded at: https://www.mdpi.com/article/10.3390/molecules27196488/s1, Table S1. *Capparis spinosa* L. metabolites annotated according to the UHPLC-QTOF-MS analysis. The annotated classified compounds are reported together with their raw abundance values and composite mass spectra (mass and abundances combination).

## Sample Availability

Samples of *C. spinosa rupestris* extract (CSE) are available from the authors.

## Abbreviations

Caco-2: human colorectal adenocarcinoma cell line; CSE: *C. spinosa* subsp. *rupestris* extract; DCFH_2_-DA: 2′,7′-dichlorodihydrofluorescein diacetate; DM: dry matter; DMNQ,2,3-dimethoxy-1,4-naphthalenedione; GAE: gallic acid equivalent; HuDe: Human Dermal fibroblast; ORAC:Oxygen Radical Scavenging Capacity; PON2:paraoxonase-2; T24: human epithelial cell line from bladder carcinoma; ROS: reactive oxygen species; TAC:Total antioxidant capacity; TE:Trolox equivalents; TP: total polyphenols; UHPLC-ESI/QTOF-MS: ultra-high-performance liquid chromatography quadrupole-time-of-flight mass spectrometry; UROtsa:immortalized human urothelium cell line.
